# The quality of antenatal care in rural Tanzania: what is behind the number of visits?

**DOI:** 10.1186/1471-2393-12-70

**Published:** 2012-07-23

**Authors:** Angelo S Nyamtema, Alise Bartsch-de Jong, David P Urassa, Jaap P Hagen, Jos van Roosmalen

**Affiliations:** 1 Department of Obstetrics and Gynecology, Saint Francis Referral Hospital, Ifakara, Tanzania; 2 Department of Medical Humanities, EMGO Institute for Health and Care Research, VU University Medical Centre, Amsterdam, The Netherlands; 3 Department of Community Health, Muhimbili University of Health and Allied Sciences, Dar es Salaam, Tanzania; 4 Department of Obstetrics, Leiden University Medical Centre, Leiden, The Netherlands

## Abstract

**Background:**

Antenatal care (ANC) provides an important opportunity for pregnant women with a wide range of interventions and is considered as an important basic component of reproductive health care.

**Methods:**

In 2008, severe maternal morbidity audit was established at Saint Francis Designated District Hospital (SFDDH), in Kilombero district in Tanzania, to ascertain substandard care and implement interventions. In addition, a cross-sectional descriptive study was carried out in 11 health facilities within the district to assess the quality of ANC and underlying factors in a broader view.

**Results:**

Of 363 severe maternal morbidities audited, only 263 (72%) ANC cards were identified. Additionally, 121 cards (with 299 ANC visits) from 11 facilities were also reviewed. Hemoglobin and urine albumin were assessed in 22% – 37% and blood pressure in 69% - 87% of all visits. Fifty two (20%) severe maternal morbidities were attributed to substandard ANC, of these 39 had severe anemia and eclampsia combined. Substandard ANC was mainly attributed to shortage of staff, equipment and consumables. There was no significant relationship between assessment of essential parameters at first ANC visit and total number of visits made (Spearman correlation coefficient, r = 0.09; p = 0.13). Several interventions were implemented and others were proposed to those in control of the health system.

**Conclusions:**

This article reflects a worrisome state of substandard ANC in rural Tanzania resulting from inadequate human workforce and material resources for maternal health, and its adverse impacts on maternal wellbeing. These results suggest urgent response from those in control of the health system to invest more resources to avert the situation in order to enhance maternal health in this country.

## Background

Antenatal care (ANC) provides an important opportunity for pregnant women with a wide range of interventions including education, counseling, screening, treatment, monitoring and promoting the well-being of the mother and fetus [1-3]. Evidence of the effectiveness of ANC interventions exists around the world when sought early in pregnancy and quality care continues until delivery [2,4,5].

Tanzania has adopted the World Health Organization (WHO) recommendation of a minimum of four goal-oriented ANC visits during a woman’s pregnancy [6,7]. At minimum blood pressure (BP), hemoglobin (Hb) estimation, weight gain, testing of urine for albumin and sugar, fundal height, fetal lie and movements/ or heart rate assessments are recommended in every ANC visit. Tests for syphilis, HIV status, blood group and Rhesus factor as well as maternal height measurement are recommended at least once. Other ANC services include preventive strategies for tetanus, malaria, anemia and mother to child HIV-transmission, education (birth preparedness and complications readiness, care of newborn, family planning as well as treatment of detected ill health.

Although the majority (94%) of pregnant women in Tanzania attend ANC at least once, the quality of care has been of great concern [8]. Maternal mortality and maternal morbidity audit (4 M study) was established at Saint Francis Designated District Hospital (SFDDH) in 2008 aimed to improve service delivery and utilization, and recommend relevant policies [9]. A second phase was carried out in December 2010 to assess in a broader view the quality of antenatal care services and underlying factors in Kilombero district, Tanzania.

## Methods

### Study area

Kilombero is a rural district located in the south-western part of Tanzania. In 2002 it had a total population of 321,661 people with annual population growth rate of 2.6% [10]. Antenatal care services are offered in 44 health facilities including a 372-bed SFDDH (including maternity waiting home services), a 120 bed capacity Illovo (parastatal) hospital, 4 public health centers and 38 private and public dispensaries scattered around the district.

### Sample size and sampling technique

The first phase of the 4 M study involved audit of all mothers with severe maternal morbidities and mortalities at SFDDH. The inclusion criteria and the auditing process have been described elsewhere [11]. The second phase of the study involved stratified sampling technique to obtain 11 health facilities out of 44 health institutions in Kilombero district (25% representation fulfilling WHO recommendation to cover at least 25-30% of the health facilities in the area when assessing quality of care [12]. These included 2 hospitals, 2 health centers and 7 dispensaries. All clients who came for antenatal clinics on the day of study were included in this study.

### Data collection

Data of the first phase (severe maternal morbidities audit) was collected and entered in Access database. The second phase of the study was a cross-sectional descriptive study which was intended at assessing the quality of antenatal care in the district. Four tools were used in this phase i.e. two checklists and two semi-structured questionnaires. The first checklist was used at the exit of clients to assess ANC cards for completeness of parameters, routinely assessed during ANC visits. These parameters included weight, maternal height, blood pressure, hemoglobin estimation, glucose in urine, albumin in urine, VDRL test, HIV test, blood group and rhesus status, provision of hematenics (iron and folate) and mebendazole. The presence of risk factor(s) which are routinely recommended on the ANC card were also reviewed. These include history of Caesarean section, age below 20 years, primigravida at age above 34 years, grand multiparous (more than 5 previous deliveries) and stature less than 150 cm. The ANC guideline in Tanzania recommends that women with these risk factors deliver in a hospital with comprehensive emergency obstetric services. An exit interview of ANC clients was carried out using a semi-structured questionnaire to assess whether they were advised on delivery and if they were satisfied with the quality of service they received. Another checklist was used to assess the staffing level, availability of essential equipment, medical supplies and drugs necessary for provision of ANC services in each health facility. A second semi-structured questionnaire was also used to interview in-charges of health facilities about the factors that affected the quality ANC service.

Ethical clearance for the study was obtained from SFDDH Research and Publication Committee. Permission to conduct the cross-sectional descriptive study was obtained from the office of the District Medical Officer and the respective in-charges of the selected health facilities. Verbal informed consent was obtained from all interviewees i.e. in-charge of health facilities and clients whose ANC cards were reviewed. Confidentiality, privacy and cultural values were also taken into consideration.

### Data analysis

Quantitative data was analyzed using SPSS software. The principal summary measures were proportions of essential parameters assessed during ANC visits and the corresponding 95% confidence intervals (95% CI). The relationship between assessment of essential parameters at first visit and the total number of visits made was determined using correlation analysis. Essential parameters (BP, Hb and albumin in urine) measured at first ANC visit were scored i.e. , each parameter was given one point when it appeared that it was assessed, making a maximum score of 3. The authors hypothesized that by assessing these parameters, involving her blood sample, urine sample and physique, a woman would feel adequately assessed and hence motivated to make more visits. Although blood and urine samples are also used for other tests, it was logically assumed that these tests may not have changed in the way how a woman felt to be assessed during the first visit. Mothers with complications of abortion, ectopic pregnancies and those who started ANC visits after 20 weeks of gestation were excluded from this correlation analysis because they were not expected to make a recommended minimum of four visits.

Qualitative data was analyzed using a method described by Graneheim and Lundman [13]. Analysis included thorough reading of the transcribed text to identify meaning units. The meaning units were then condensed, abstracted, coded and then categorized according to similarities and differences in content.

## Results

### Findings from the audit phase

Of all 363 women with severe maternal morbidities admitted at SFDDH from October 2008 to July 2010, ANC cards were found in only 263 (72%). Of the women without ANC cards some left them at home, others had not started ANC visits and the rest were misplaced within the hospital. Out of the total 754 ANC visits made by these 263 women with antenatal cards, BP, Hb and albumin in urine were assessed in only 69%, 25% and 22% respectively. The audit team attributed 52 (20%) of the 263 mothers with ANC cards to substandard antenatal care. Of these 39 (75%) were mothers presenting with severe anemia in pregnancy and eclampsia combined who had attended ANC clinics regularly but the respective parameters were never checked. During audit it was not clear whether the substandard assessment of these parameters was due to poor supply of essential ANC equipment, drugs and consumables or because of poor performance of care providers. Correlation analysis indicated that there was no significant relationship between the assessment of essential parameters at first visit and the total number of ANC visits made by 63 women who qualified for analysis (Spearman correlation coefficient, r = 0.09; p = 0.13).

### Findings from cross sectional descriptive study

Antenatal cards belonging to 121 pregnant women attending in 11 antenatal clinics in Kilombero district were reviewed with an average of 11 (ranging from 4 – 35) cards from each facility. Of these cards, 95 (79%) belonged to women with primary education, only one belonged to a client with post secondary education. Of all women 107 (88%) were peasants, 7 (6%) petty businesswomen and 7 (6%) were either employee in the public or nongovernmental institutions. More than a quarter (26%) of these women were primigravida, 51% were gravida 2–4 and 92% were married.

Out of the total 299 ANC visits made hemoglobin estimation, glucose and albumin in urine were assessed in almost one third (27% – 37%) of the visits (Table 1). Although majority (83%) of mothers had made at least 2 ANC visits, blood group and rhesus status, and VDRL were tested in only 7% and 48% respectively (Table 2). With exception of Hb estimation, HIV test, blood grouping and rhesus status, and provision of mebendazole, the rest of the routine services recommended for ANC in Tanzania were statistically significantly more checked / provided in the hospitals than in lower health facilities.

**Table 1:** Proportions of check ups of parameters and prophylactic drugs recommended for every ANC visit

**Parameters**	Hospitals* (ANC visits = 127)		**First Level Health Facilities (ANC visits = 172)**		**Total% (visits = 299)**
	**%**	**95% CI**	**%**	**95% CI**	
Weight	89	84 - 94	74	67 - 81	80
Blood pressure	98	96 - 99	78	72 - 84	87
Hb estimation	45	34 - 54	32	25 - 39	37
Albumin in urine	47	38 - 56	20	14 - 26	32
Glucose in urine	42	33 - 51	15	10 - 20	27
Iron tablets	77	70 - 84	45	38 - 54	59
Folate tablets	71	63 - 79	45	38 - 54	56

NB: *Only data from the second phase of the study (cross sectional descriptive study) is included here; First Level Health Facilities = health centers & dispensaries.

**Table 2:** Proportions of check ups of parameters and prophylactic drugs recommended at least once during ANC period

**Parameters**	**Hospitals Clients = 43**		**First Level Health Facilities Clients = 78**		**Total clients n = 121**
	**%**	**95% CI**	**%**	**95% CI**	**%**
Height	93	88 - 99	56	45 - 67	69
HIV Test	91	82 - 99	74	64 - 84	80
VDRL Test	67	53 - 81	37	26 - 48	48
Blood group & Rhesus factor	5	1 - 12	9	3 - 15	7
Mebendazole	77	64 - 90	54	43 - 65	62

NB: Only data from the second phase of the study (cross sectional descriptive study) is included; First Level Health Facilities = dispensaries and health centers.

Of all women 63 (52%) had at least one risk factor. Of women with risk factors 27 (42%) were under 20 years of age, 12 (19%) had short stature less than 150 cm and 11 were grand multiparous. Delivery advice was provided to only 40 (33%) women attending ANC on the day of study. The most frequent delivery advice (93%) given to women with risk factors was hospital delivery, when to go and use of maternity waiting home. On the other hand, 25 (40%) women with risk factors reported that they did not receive any advice on the delivery plan. On the contrary, 93 (77%) women reported that they were satisfied with the ANC services they received in these facilities. This number included women who had a risk factor but never received any delivery advice.

### Factors affecting ANC services delivery

BP machines, stethoscopes, weighing scales, HIV test kits, folic acid, mebendazole and SP drugs for IPT were available in nearly all (91% – 100%) facilities during the period of this study. Hb estimation machines were available in less than two thirds (64%) of the health facilities. The respondents (in charge of health facilities) reported that some essential equipment like BP machines were of poor quality leading to short durability contributing to the shortage. Glucostik and albustik kits were available only in 18% and 27% of all health facilities respectively. Hb estimation machines, Glucostik and albustik kits were completely unavailable in these facilities for up to 12 months before the study. Generally, there was severe shortage of staff for antenatal care in all dispensaries and health centers. Shortage of qualified staff and irregular supply of essential equipment, drugs and consumables were considered by 91% and 64% of the respondents respectively as the major underlying factors for substandard ANC (Table 3).

**Table 3:** Factors affecting quality antenatal care in Kilombero district

**Factors affecting quality antenatal care**	**Proportions of respondents n = 11**
Shortage of qualified staff	91%
Irregular supply of ANC equipment and drugs	64%
Regular but inadequate supplies	45%
Cultural factors and ignorance among pregnant women	36%
Lack of staff motivation	27%
Poor infrastructure for ANC	18%
Long distance to the health facility with ANC services	9%

### Interventions

A list of strategic interventions for quality ANC improvement in the district were proposed and implemented as a result of audit. These included regular feedback to ANC providers, recommendations were shared with the regional and district heath authorities to improve staffing levels, essential supplies and equipment, and supportive supervision for quality antenatal care. The Medical Store Department (MSD), the central government supplier of medical equipment, drugs and consumables, was also contacted through a series of meetings and advised to improve the ordering and supply mechanisms to enhance quality and availability of essential supplies.

## Discussion

This article reveals a state of poor assessment of essential parameters for antenatal care in rural Tanzania despite adoption of goal-oriented ANC with a limited number of visits. Despite the complexity of interacting factors, antenatal education, screening and treatment of common causes of maternal and perinatal mortalities and morbidities such as pre-eclampsia and anemia constitute a body of benefits of ANC interventions [5,14,15]. The effectiveness of such ANC interventions has been linked to its quality, access and coverage [15]. The fact that comparable findings indicating substandard provision of vital ANC services have been repeatedly reported in rural sub-Saharan Africa since the last decade, suggest dormant health systems, an alarming state of inadequate utilization of research findings in these countries and the urgent need for improvement [16-20].

The fact that 20% of severe maternal morbidities were attributed to substandard ANC suggests a remarkable proportion of adverse pregnancy outcome that could be reduced by improving this program. These findings suggest also that women from rural Tanzania belong to a group that would need quality ANC the most in the world [14]. The high prevalence of ANC attendance in sub-Saharan Africa [8,21] and positive attitude about antenatal care despite its poor quality [22], offer an important opportunity for quality maternal care improvement. These findings pose a great challenge to those in control of health systems in sub-Saharan Africa to invest more resources in antenatal care in order to accelerate maternal health in rural areas.

As opposed to the authors’ hypothesis there was no significant relationship between assessment of essential parameters of pregnant women at the first ANC visit and total number of visits. These results could partly be explained by lack of alternative of these women and the presence of high degree of ignorance which was manifested by high satisfaction in the circumstance of poor quality of ANC services. These findings suggest also that improvement of ANC may not necessarily improve attendance in this region.

Health care providers attributed substandard ANC to irregular supply of essential equipment and drugs, poor infrastructure for ANC and shortage of staff. The fact that Hb was estimated in only 37% of ANC visits despite Hb estimation machines being available in 64% of health facilities raise more questions on the performance, accountability, commitment of health care providers and supervision in the health sector. Our findings recommend more resources to ensure regular essential supplies, drugs and equipment, train more skilled staff and carry out supportive supervision in order to improve ANC services [14,16].

### Limitations

The statistical analysis assumed that all observations were independent. However, in a first level health facility (dispensary or health center) there are usually few health workers who perform ANC. These are likely to repeat mistakes or deliver good care on all mothers they attend in ANC. Thus, consultations by the same health worker may not necessarily be independent from each other, and even more so if some equipment is missing. *Validity of ANC card data:* it was assumed that all data in the ANC cards were true. However, some tests may have been done, but not recorded; some tests may have been recorded, but not done. It was not possible to cross-check through the interview with mothers whether all these parameters were performed or not. The fact that satisfaction to ANC services is subjective, the results posed a potential limitation. Satisfaction can be influenced by a number of factors including knowledge on the required types of services and attitude of the individual clients. Based on these factors, clients might have expressed different levels of satisfaction even if they received similar services.

## Conclusions

This article reflects a worrisome state of substandard antenatal care in rural Tanzania resulting from complex interacting factors including persistent lack of skilled human and material resources as well as irresponsive leadership in the health sector. Findings from this audit program suggest substantial adverse impact on maternal wellbeing resulting from poor quality of ANC. These results suggest urgent response from those in control of the health system to invest more resources in antenatal care to avert the situation and enhance maternal health in Tanzania.

## Abbreviations

ANC, Antenatal care; CI, Confidence intervals; SFDDH, Saint Francis designated district hospital.

## Competing interests

The authors declare that they have no competing interests.

## Authors’ contributions

ASN, ABJ, JP and JvR designed and took part in the audit process and data analysis. DPU contributed to writing of the manuscript. All authors read and approved the final manuscript.

## Pre-publication history

The pre-publication history for this paper can be accessed here:

http://www.biomedcentral.com/1471-2393/12/70/prepub
